# The augmented physician: AI and the future of clinical cognition

**DOI:** 10.3389/frai.2026.1744544

**Published:** 2026-02-23

**Authors:** Khayreddine Bouabida, Breitner Gomes Chaves, Enoch Anane

**Affiliations:** 1 UConn Health, Farmington, CT, United States; 2 Connecticut Children's Medical Center, Hartford, CT, United States; 3 Research Center of the University of Montreal Hospital Centre, Montreal, QC, Canada; 4 Department of Community Health Sciences, Université de Sherbrooke, Sherbrooke, QC, Canada; 5 UMass Chan Medical School, Worcester, MA, United States

**Keywords:** artificial intelligence (AI), clinical cognition, clinical decision support, medical education, patient-centered care, physician burnout

## Abstract

Medicine stands at a cognitive tipping point as the volume of biomedical information expands faster than clinicians can realistically monitor, synthesize, and apply new evidence in routine practice. Once a marker of scientific progress, this acceleration now challenges the foundations of clinical expertise, patient safety, and medical education. This Perspective examines the widening gap between evidence generation and evidence implementation, arguing that artificial intelligence should not replace clinicians but serve as a cognitive partner. Properly designed and ethically governed systems can assist clinicians by organizing and contextualizing large bodies of information, enabling greater focus on clinical judgment, empathy, and human connection. When integrated thoughtfully, artificial intelligence has the potential to strengthen patient engagement, reduce administrative burden, and support shared human and machine cognition in care delivery. Sustaining clinical excellence in an era of accelerating information growth will depend on embracing artificial intelligence as a collaborative tool and redefining how physicians learn, think, and care. The future of medicine will remain profoundly human, precisely because it is intelligently augmented.

## Introduction

This article is a reflective Perspective on one of medicine’s most underestimated challenges: the rapid expansion of biomedical information and its cognitive implications for clinicians. As the volume of research publications, clinical data, and digital health outputs grows, physicians are increasingly required to navigate an information environment that no individual can realistically monitor or synthesize alone. The traditional model of physician expertise, rooted in memorization, manual synthesis, and lifelong recall, has reached its practical limits within this evolving ecosystem (Densen, 2011; Kelly et al., 2019; MacIntyre et al., 2023).

Historically, the growth of medical knowledge was sufficiently gradual to allow clinicians to remain current through conventional educational pathways. In 1950, the body of indexed medical information was estimated to double approximately every 50 years. By 1980, this interval had shortened to about seven years, and by 2010 to roughly 3.5 years. Contemporary estimates suggest that the volume of biomedical information and published literature now expands even faster and is often described as doubling within months (Densen, 2011; Kelly et al., 2019; Balas and Boren, 2000; Topol, 2019; Mir et al., 2023). Importantly, this acceleration reflects the proliferation of data, publications, and subspecialty evidence rather than the rapid replacement of foundational clinical principles, many of which remain stable over decades of practice.

Even so, the consequences for clinical cognition are substantial. Clinicians must continually evaluate new evidence, guideline updates, subgroup analyses, and context-specific findings layered onto existing knowledge. This expanding informational periphery contributes to a widening gap between evidence generation and evidence implementation, challenging clinicians’ ability to deliver consistently evidence-informed care while preserving the relational and ethical dimensions central to medicine (Densen, 2011; Kelly et al., 2019; Balas and Boren, 2000; Topol, 2019; Mir et al., 2023).

This Perspective explores how artificial intelligence should be understood not as a replacement for physicians, but as a cognitive partner within this increasingly complex information landscape. Rather than automating judgment or supplanting expertise, artificial intelligence can assist clinicians in organizing, contextualizing, and translating large volumes of biomedical information into clinically relevant insights. Framed as an instrument of cognitive augmentation rather than epistemic authority, artificial intelligence emerges as a necessary component for preserving clinical excellence, patient safety, and ethical decision making in modern medical practice (Densen, 2011; Kelly et al., 2019; Balas and Boren, 2000; Topol, 2019; Mir et al., 2023).

## The unequal burden of information overload

The cognitive burden imposed by the contemporary biomedical information environment is not distributed evenly across medical specialties. Certain fields face disproportionate exposure to information overload because of the intensity, breadth, and time sensitivity of their clinical responsibilities. Emergency medicine, primary care, and high-complexity specialties illustrate how information overload translates directly into clinician distress and patient safety risk.

Emergency medicine physicians are particularly vulnerable. A multisite National Health Service study across four hospital trusts in Northern England found that most emergency physicians perceived information overload as a serious and worsening problem. Contributors included expectations of constant availability, excessive email volume, and dense multidisciplinary communication. Reported consequences included guideline fatigue, prolonged working hours, increased stress, and impaired decision making (Sbaffi et al., 2020). These findings align with a meta-analysis showing that approximately 40% of emergency physicians experience high levels of emotional exhaustion and depersonalization, with emergency medicine physicians more susceptible to burnout than physicians in many other specialties (Zhang et al., 2020).

Primary care physicians face a different but equally overwhelming burden driven more by scope than acuity. A qualitative study published in *JAMA Internal Medicine* described primary care work as insurmountable, overwhelming, and undoable. Physicians reported that specialists routinely defer medication refills, prior authorizations, test results, and diagnostic follow-up to them, creating a “giant funnel” effect that shifts responsibility for data management and coordination to primary care (Agarwal et al., 2020). One physician noted that clinical work increasingly involves managing data, numbers, and processes rather than caring for people. National data from *JAMA Network Open* show that primary care physicians had the highest burnout rates among all health care occupations between 2018 and 2023, ranging from 46.2 to 57.6% (Mohr et al., 2025).

Specialty-specific analyses reveal additional vulnerabilities. A national study of 3,588 United States resident physicians found significantly higher burnout rates in urology, neurology, emergency medicine, ophthalmology, and general surgery compared with internal medicine, with relative risks ranging from 1.23 to 1.48 (Dyrbye et al., 2018). Burnout also has direct implications for patient safety. A large systematic review and meta-analysis demonstrated that physician burnout is associated with more than double the odds of patient safety incidents and low professionalism, with the strongest association observed in emergency medicine (Hodkinson et al., 2022).

Taken together, these findings indicate that information overload is not an abstract cognitive concern, but a specialty-dependent threat to clinician well-being and patient safety. They suggest that artificial intelligence-enabled cognitive support may be most urgently needed in emergency medicine, primary care, and other high-complexity clinical environments where information overload directly compromises decision quality and care delivery.

## The collapse of the medical knowledge timeline

The pace at which biomedical information is generated and disseminated has shifted from a manageable evolution to an unprecedented expansion. In 1950, the volume of indexed medical literature was estimated to double roughly every 50 years, allowing many physicians to complete a career without facing a fundamentally altered information landscape. By 1980, this interval had shortened to approximately seven years, and by 2010 to about 3.5 years. Contemporary estimates suggest that the volume of biomedical publications and digital medical data continues to expand at a rapid pace and is often described as doubling within months (Densen, 2011; Kelly et al., 2019; Balas and Boren, 2000; Topol, 2019; Mir et al., 2023).

It is important to distinguish this acceleration in information volume from changes in validated clinical knowledge. The rapid growth of publications does not imply that foundational principles of diagnosis and treatment are overturned at the same rate. Much of this expansion occurs at the periphery of medical practice, including incremental refinements in subspecialty care, studies of variable quality, exploratory findings, and data relevant to narrowly defined patient populations. For many clinicians, the core frameworks guiding common conditions such as hypertension, diabetes, or acute chest pain remain stable over long periods of practice.

Nevertheless, the expansion of the informational environment has profound implications for daily clinical work. A medical student entering training in 2020 will encounter a literature base that grows substantially across medical school and residency, even if core clinical principles remain relatively consistent. Clinicians are expected to evaluate new trials, updated guidelines, subgroup analyses, and emerging safety signals layered onto established knowledge. Traditional mechanisms of knowledge transfer, including textbooks, lectures, and episodic continuing medical education, are poorly suited to this continuous evaluation process. The result is a widening gap between the generation of evidence and its consistent application in practice (Densen, 2011; Balas and Boren, 2000).

In earlier eras, professional expertise was largely defined by the depth of an individual physician’s internalized knowledge. In the current environment, expertise is increasingly defined by the ability to navigate, interpret, and operationalize complex information systems that exceed individual cognitive capacity. The collapse of the medical knowledge timeline reflects not an erosion of clinical fundamentals, but a transformation in how medical knowledge must be accessed, filtered, and applied. This shift signals the need for new tools, new learning models, and new forms of cognitive partnership to sustain high-quality care (Densen, 2011; Kelly et al., 2019; Balas and Boren, 2000; Topol, 2019; Mir et al., 2023).

## The impossible cognitive burden

Even the most dedicated physician cannot realistically keep pace with the contemporary biomedical information environment. Clinicians manage numerous patient encounters each day, each requiring the application of established clinical principles together with selective integration of new evidence, updated guidelines, and context-specific considerations. At the same time, thousands of biomedical manuscripts are published daily across journals, preprint servers, and clinical trial registries (Balas and Boren, 2000; Topol, 2019). Although much of this literature does not alter routine practice, clinicians are expected to remain alert to findings that may affect patient safety, therapeutic effectiveness, or standards of care.

This imbalance has contributed to a widening knowledge-practice gap that reflects structural difficulty rather than individual incompetence. Classic analyses suggest that validated evidence may take more than a decade to become consistently incorporated into clinical care (Balas and Boren, 2000). During this lag, patients may receive care that is concordant with existing standards yet misaligned with the most current evidence, highlighting system-level limitations rather than individual neglect.

The burden of this environment extends beyond clinical accuracy to clinician well-being. Physicians navigating dense documentation requirements, expanding quality metrics, and evolving evidence bases report high levels of cognitive fatigue, burnout, and moral distress, particularly when aware of emerging data they lack the time or tools to evaluate fully. National data and specialty-specific analyses demonstrate that burnout affects a substantial proportion of clinicians, with rates exceeding 40% in several frontline specialties and strong associations with reduced professional engagement and patient safety incidents (Mohr et al., 2025; Dyrbye et al., 2018; Hodkinson et al., 2022). Administrative and regulatory demands compete directly with clinical reasoning and patient interaction, further straining limited cognitive resources.

Expecting unaided human cognition to manage this expanding informational landscape is no longer sustainable. The challenge confronting medicine is not one of insufficient intelligence or motivation, but one of cognitive bandwidth. To protect both patients and clinicians, medical practice must evolve from a model centered on individual memory and manual synthesis to a model supported by systems that assist with evidence filtering, prioritization, and contextualization. Properly designed and ethically governed artificial intelligence offers a scalable means of supporting this transition toward system-assisted cognition (Kelly et al., 2019; Topol, 2019; Sahni and Carrus, 2023; Dave et al., 2025; Lim et al., 2026).

## AI as a cognitive extension and partnership, not a replacement

The solution to medicine’s growing cognitive burden cannot rest on increased individual effort alone. It requires systemic augmentation. Artificial intelligence offers such augmentation not as a replacement for physicians, but as an extension of human cognition designed to support clinical reasoning within an increasingly complex information environment.

In this perspective, artificial intelligence is used as an umbrella term encompassing a range of technologies with distinct functions and levels of maturity. These include supervised machine learning models embedded in electronic health records for risk prediction, clinical decision support algorithms, automated documentation and scribing tools, large language models (LLMs) capable of summarizing medical literature, and foundation models trained on multimodal clinical data. While these systems differ substantially in implementation, adoption, and risk profiles, they share a common function: assisting clinicians in organizing, prioritizing, and contextualizing information at a scale that exceeds individual cognitive capacity (Kelly et al., 2019; Topol, 2019; Sahni and Carrus, 2023).

Across these applications, artificial intelligence supports clinical practice by filtering large bodies of literature, identifying relevant guideline updates, flagging safety signals, and synthesizing patient-specific data into interpretable outputs. In doing so, AI functions not as an autonomous decision maker, but as cognitive scaffolding that enhances situational awareness and supports more informed human judgment. This reframes artificial intelligence as a tool for knowledge translation rather than knowledge generation, directly addressing the widening gap between evidence production and bedside implementation (Kelly et al., 2019; Balas and Boren, 2000; Topol, 2019).

The central question, therefore, is not whether artificial intelligence can replace clinicians, but whether contemporary medicine can function safely without system-assisted cognitive support. In an environment characterized by expanding subspecialty literature, regulatory complexity, and administrative burden, clinicians who rely solely on manual information processing face increasing difficulty maintaining situational awareness across evolving evidence landscapes. This reflects not diminished competence, but an information ecosystem that has outgrown unaided cognitive strategies.

At the same time, artificial intelligence systems operating in isolation are inherently limited. Algorithms lack contextual understanding, moral reasoning, and the capacity for empathy. They are vulnerable to bias introduced through training data and may perform inconsistently across populations and clinical settings (MacIntyre et al., 2023; Sahni and Carrus, 2023). The most robust model for the future of medicine is therefore a human–AI partnership in which machines contribute speed, scale, and pattern recognition, while clinicians provide judgment, ethical oversight, and accountability for final decisions.

Within this partnership, the physician is not diminished but amplified. Artificial intelligence enables clinicians to access relevant information more efficiently, reducing time spent searching for data and increasing time available for interpretation, communication, and shared decision making. In this sense, AI strengthens rather than undermines the therapeutic relationship by allowing clinicians to focus more fully on the human dimensions of care. As described by Topol and others, this convergence represents not the end of the physician’s role, but its evolution toward an augmented form of clinical expertise grounded in both technological support and human values (Densen, 2011; Kelly et al., 2019; MacIntyre et al., 2023; Balas and Boren, 2000; Topol, 2019).

## Empirical evidence that AI can reduce cognitive burden and burnout

Beyond conceptual promise, a growing body of empirical evidence demonstrates that artificial intelligence can meaningfully reduce documentation burden, cognitive load, and clinician burnout when implemented as a clinician supervised tool. Recent systematic reviews and multicenter studies provide early but compelling support for AI driven workflow augmentation across diverse clinical settings.

A 2025 systematic review and meta analysis found that artificial intelligence assisted documentation tools were associated with a moderate reduction in documentation workload and burnout when clinicians reviewed and edited AI generated drafts, with a standardized mean difference of −0.72 (Zhao et al., 2025). These findings indicate that AI functions most effectively not as an autonomous system, but as a supportive drafting and organizational aid embedded within existing clinical workflows.

Evidence from real world implementation further supports this conclusion. In a multicenter quality improvement study involving 263 physicians, the introduction of an ambient artificial intelligence scribe was associated with a significant reduction in burnout from 51.9 to 38.8% after 30 days of use. Clinicians also reported improvements in cognitive task load and an average reduction of nearly one hour per day in after hours documentation (Olson et al., 2025). Importantly, these gains were achieved without reducing clinician oversight or responsibility for final documentation.

Additional data from *JAMA Network Open* demonstrate that clinicians using artificial intelligence powered documentation platforms experienced significantly reduced cognitive demand and documentation effort, alongside perceived improvements in clinical efficiency and patient centered care (Stults et al., 2025). These findings are particularly salient given that physicians now spend more than 50% of their working time interacting with electronic health records, a well documented contributor to burnout and professional dissatisfaction (Sahni and Carrus, 2023).

Broader scoping reviews further reinforce these observations. Across studies examining natural language processing, AI integrated electronic health records, clinical decision support systems, and generative AI tools, reductions in administrative burden and improvements in job satisfaction were consistently reported, with burnout emerging as the most frequently addressed mental health outcome (Dave et al., 2025). In diagnostic specialties, artificial intelligence assisted interpretation has been shown to substantially reduce interpretation time for abnormal imaging and laboratory findings without compromising diagnostic accuracy (Lim et al., 2026).

Critically, these efficiency gains do not appear to trade speed for safety. Generative artificial intelligence based electronic medical record systems reduce documentation time by approximately 40%, while voice recognition and AI scribing technologies reduce charting time by nearly 30%, resulting in overall administrative burden reductions exceeding 30% (Lim et al., 2026). Across studies, these benefits consistently depend on clinician review and oversight, reinforcing the central premise that artificial intelligence functions most effectively as cognitive augmentation rather than autonomous decision making.

## AI, patient engagement, and the future of patient-centered care

Concerns are often raised that increasing reliance on artificial intelligence may distance clinicians from patients or erode the relational foundations of care. However, when integrated thoughtfully, artificial intelligence has the potential to strengthen patient engagement and reinforce patient centered care by reshaping how clinical time and attention are allocated (Balas and Boren, 2000; Sbaffi et al., 2020; Zhang et al., 2020).

Evidence from telehealth, digital health platforms, and clinical decision support systems suggests that these tools can improve access to care, particularly for patients facing geographic, mobility, or scheduling barriers. By streamlining documentation, automating routine administrative tasks, and supporting rapid information retrieval, artificial intelligence can reduce nonclinical workload and allow clinicians to devote greater attention to direct communication with patients, families, and caregivers. This redistribution of effort creates space for shared decision making, education, and emotional presence, dimensions of care that are frequently constrained in contemporary practice (Balas and Boren, 2000; Topol, 2019; Sahni and Carrus, 2023).

Artificial intelligence also enables patients to take a more active role in their own care. Remote monitoring systems, personalized digital feedback tools, and algorithm supported care pathways can provide patients with timely and understandable information about their health status and treatment options. When governed appropriately, these tools enhance patient understanding, self management, and engagement without supplanting the clinician’s role. Rather than reinforcing hierarchical models of care, they support collaborative relationships in which information and responsibility flow in both directions.

These changes affect not only physicians but also nurses and other health professionals whose workflows are deeply intertwined with documentation, coordination, and patient communication. AI enabled tools that reduce repetitive charting and information retrieval can free nursing and interprofessional teams to spend more time on direct patient care, education, and advocacy, thereby strengthening team based, patient centered practice.

Crucially, the impact of artificial intelligence on patient engagement depends on how it is implemented. Systems designed to support transparency, explanation, and dialogue can deepen trust and understanding, while poorly designed tools risk confusion or detachment. When guided by ethical oversight and clinician leadership, artificial intelligence represents not a threat to patient centered care, but an opportunity to strengthen it by improving accessibility, continuity, and the quality of clinician patient interaction (Sbaffi et al., 2020; Zhang et al., 2020).

## Ethical and educational imperatives

The integration of artificial intelligence into medicine introduces both significant opportunity and substantial responsibility. While algorithmic systems can assist clinicians by processing information at scale, they also reflect the biases, gaps, and structural inequities present in the data on which they are trained (MacIntyre et al., 2023). For this reason, artificial intelligence must be developed and implemented under rigorous standards of transparency, accountability, and equity. Clinical responsibility cannot be delegated to machines. Physicians remain accountable for interpreting algorithm-supported insights within clinical, ethical, and social contexts.

Ethical implementation alone, however, is insufficient. Medicine must also confront the reality that prevailing educational models are increasingly misaligned with the contemporary information environment. Training structures that emphasize memorization and recall were developed for an era in which knowledge evolved gradually and predictably. They are poorly suited to a clinical landscape characterized by continuous evidence generation, heterogeneous data quality, and rapid guideline evolution (Densen, 2011; Kelly et al., 2019; MacIntyre et al., 2023). Preparing physicians for this environment requires a shift from knowledge accumulation toward skills in evidence appraisal, contextual reasoning, and information stewardship. This reframing places lifelong learning at the center of professional identity, with clinicians expected to continually reassess evidence, update practices, and adapt to new information throughout their careers.

This shift demands a revised educational paradigm that prioritizes data literacy, critical thinking, and familiarity with artificial intelligence–enabled tools as an extension of digital competence. AI literacy builds on foundational digital skills by enabling clinicians to understand how algorithms are trained, how bias can emerge, and how outputs should be interpreted rather than accepted uncritically. Importantly, artificial intelligence literacy should not aim to turn clinicians into engineers, but to equip them with sufficient understanding to evaluate system limitations, recognize failure modes, and maintain clinical autonomy (Kelly et al., 2019; MacIntyre et al., 2023; Sahni and Carrus, 2023).

A related ethical challenge is the risk of automation bias. When algorithm-generated recommendations are delivered with speed and apparent authority, clinicians may defer judgment prematurely or fail to apply necessary skepticism. Preventing this requires systems designed to encourage reflection rather than passive acceptance, including explainable outputs, uncertainty signaling, and workflows that preserve deliberate human oversight. Education must reinforce these habits to ensure that artificial intelligence augments rather than erodes diagnostic reasoning.

Finally, equity must remain central to artificial intelligence deployment. High-resource institutions may rapidly benefit from advanced systems, while lower-resource settings risk exclusion due to infrastructure limitations. If artificial intelligence becomes a determinant of care quality, unequal access could widen existing health disparities. Ensuring that these technologies function as equalizers rather than stratifiers will require investment in inclusive datasets, open standards, public governance, and models designed to perform across diverse populations rather than exclusively in data-rich environments (MacIntyre et al., 2023; Sahni and Carrus, 2023). In support of this need for caution, a recent systematic review identified 19 major barriers to artificial intelligence implementation across technical, stakeholder, and societal domains, including limited explainability, workflow integration challenges, liability concerns, and uncertainty regarding cost-effectiveness, underscoring that ethical adoption requires more than technical capability alone (Li et al., 2023).

## Conclusion

Medicine has reached a cognitive inflection point. The rapid expansion of biomedical information, once a hallmark of scientific progress, now challenges the limits of unaided human cognition. The central issue facing clinicians is no longer simply what they know, but how they access, evaluate, and apply relevant evidence within an increasingly complex information environment.

Artificial intelligence offers not an escape from this challenge, but a navigational aid through it. When used as a cognitive partner, artificial intelligence can support clinicians in translating large and evolving bodies of information into contextually relevant insights while preserving human judgment, ethical reasoning, and relational care. Importantly, advances in telehealth and digital health demonstrate that thoughtfully implemented artificial intelligence can also strengthen patient engagement and patient-centered care by restoring time and attention to communication, shared decision making, and trust.

Failing to engage with this partnership risks leaving clinicians increasingly constrained by systems that evolve faster than individual cognitive strategies can adapt. The defining question for modern medicine is therefore not whether artificial intelligence will influence healthcare, but whether clinicians will actively shape its integration or passively respond to it. The most ethical and sustainable future lies not in replacement, but in collaboration, one in which machines extend the reach of human cognition and physicians remain responsible for meaning, values, and care.

The cognitive edge confronting medicine is not a threat to professional identity, but an opportunity to redefine it. By embracing artificial intelligence as a supportive tool rather than an epistemic authority, medicine can remain both scientifically rigorous and deeply human. The future of medicine will remain human, precisely because it is intelligently augmented.

## Data Availability

The original contributions presented in the study are included in the article/supplementary material, further inquiries can be directed to the corresponding author.
